# Supplementary material for: An extended reconstruction of human gut microbiota metabolism of dietary compounds
Source: Nat Commun. 2021 Aug 5;12:4728. doi: 10.1038/s41467-021-25056-x (PMC8342455; doi:10.1038/s41467-021-25056-x)
Supplement: Supplementary file 1 — Supplementary Information [file 41467_2021_25056_MOESM1_ESM.pdf]

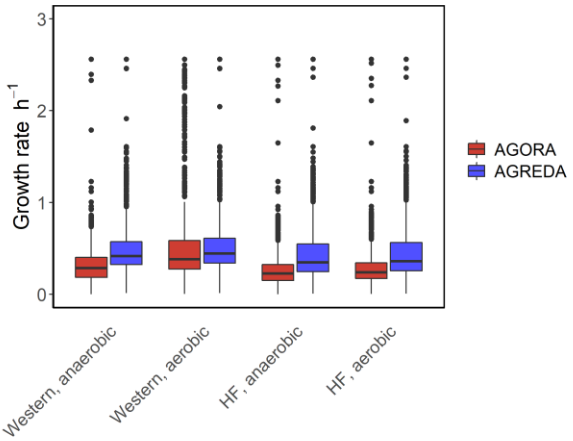

Supplementary Figure 1. Boxplot of maximum aerobic/anaerobic growth rate obtained for the different organisms present in AGREDA (blue) and AGORA (red) under two different growth medium conditions: Western and High-Fiber (HF) Diet. Maximum uptake fluxes and nutrient availability for Western and HF diets were taken from Magnusdottir et al. 2017[1]. Bottom and top of the boxes denote the first and third quartiles, respectively, and whiskers represent the values within 1.5 interquartile range above and below the box. Center line represents the median value. n=818 independently assessed organisms present in AGORA.

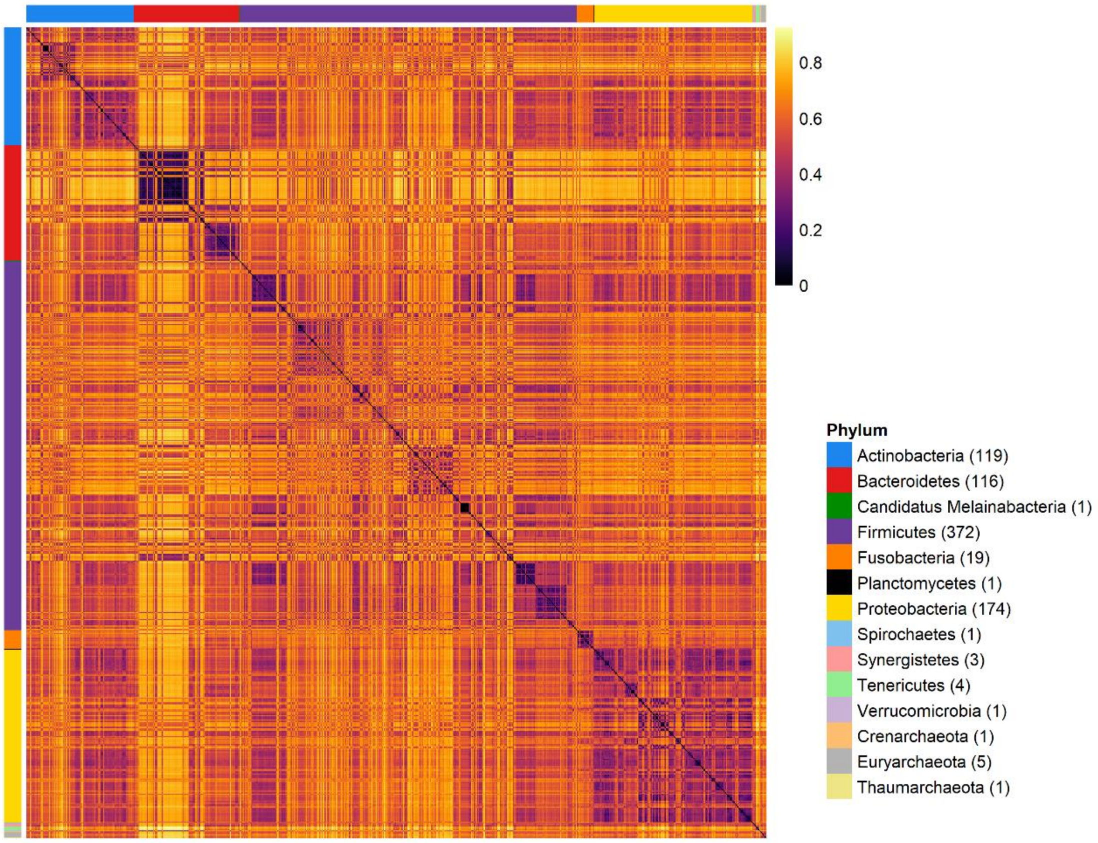

Supplementary Figure 2. Metabolic distance between different species involves in AGREDA. The metabolic distance between two species was determined with the Jaccard's distance. Metabolic models with identical set of reactions have a null metabolic distance, while completely different reconstructions have a metabolic distance of 1. Different species are sorted by phyla.

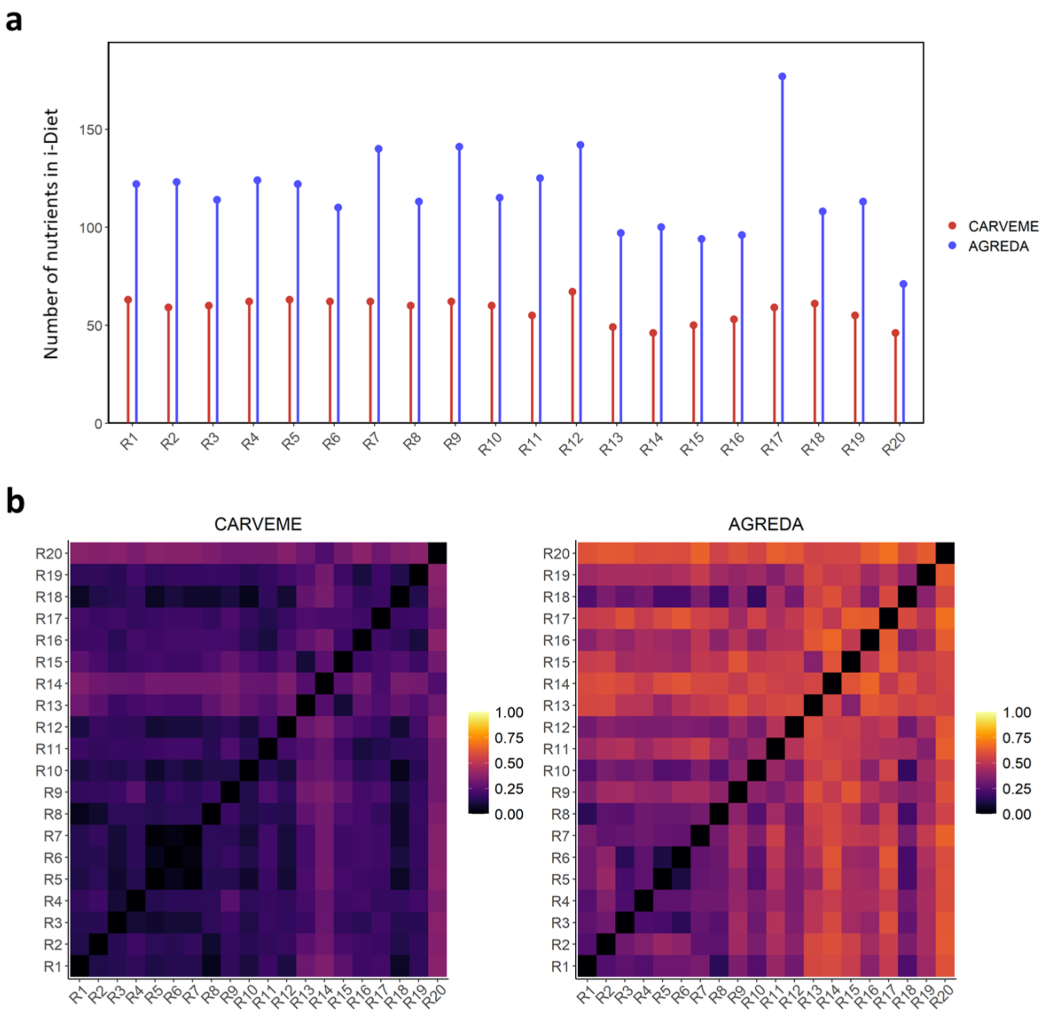

Supplementary Figure 3. Nutritional composition of 20 representative recipes of the Mediterranean diet in AGREDA and CARVEME. (a) The number of input dietary nutrients that CARVEME (red) and AGREDA (blue) are able to capture for different recipes (R1, R2,…, R20). Note that all the metabolites present in CARVEME are also included in AGREDA. **(b)** Differences between the nutritional content of the recipes captured by CARVEME and AGREDA, respectively. The Jaccard´s distance between the composition of the recipes is represented.

a

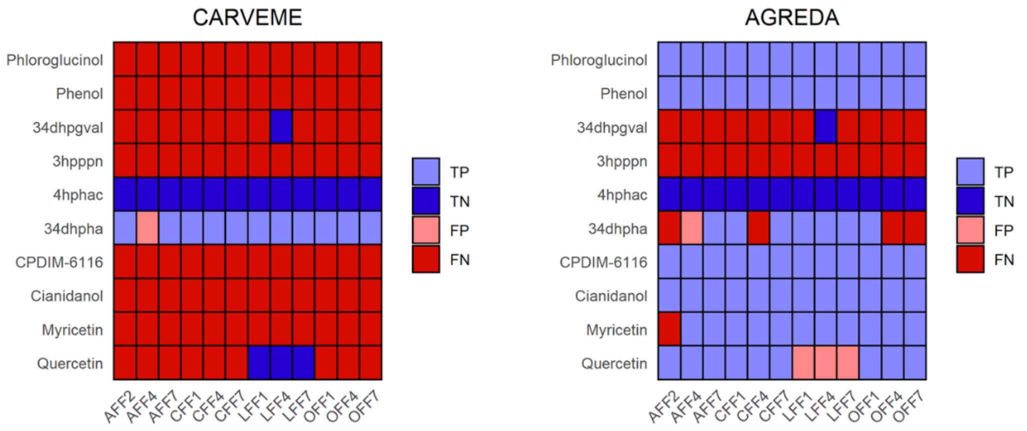

b

**Output metabolites**		**CARVEME**		**AGREDA**	
		**+**	**-**	**+**	**-**
***In vitro* experiment**	**+**	11	92	75	28
	**-**	1	16	4	13

	**CARVEME**	**AGREDA**
**Sensitivity**	0.107	0.728
**Specificity**	0.941	0.765
**Accuracy**	0.225	0.733
**Fisher´s p-value**	1	1.55×10^{-4}

Supplementary Figure 4. In-vitro experimental comparison of the predictions by AGREDA and CARVEME. (a) Comparison of AGREDA and CARVEME for predicting the presence (positive) or absence (negative) of 10 output microbial compounds derived from the fermentation of lentils with children faeces and measured with a targeted metabolomics approach. **(b)** Confusion matrix and statistical details of the comparison shown in (a). Sensitivity was determined as TP/(TP+FN), Specificity as TN/(FP+TN) and Accuracy as (TP+TN)/(TP+TN+FP+FN). The reported Fisher's p-value was two-sided. Abbreviations: 34dhpgval (5-(3',4'-Dihydroxyphenyl)-gamma-valerolactone), 3hpppn (3-(3-hydroxy-phenyl)propionate), 4hphac (4-hydroxyphenylacetate), 34dhpha ((3,4-dihydroxyphenyl)acetate), CPDIM-6116 (Dihydrocaffeic acid); 'AFF2', 'AFF4' and 'AFF7' denote samples 2, 4 and 7 of children allergic to cow's milk, respectively; 'CFF1', 'CFF4' and 'CFF7' denote samples 1, 4 and 7 of celiac children, respectively; 'LFF1', 'LFF4' and 'LFF7' denote samples 1, 4 and 7 of lean children, respectively; 'OFF1', 'OFF4' and 'OFF7' denote samples 1, 4 and 7 of obese children, respectively; TP (True Positives), TN (True Negatives), FP (False Positives), FN (False Negatives).

Subsystem	N
Flavonoid metabolism	200
Aromatic compound degradation	53
Anthocyanin biosynthesis	53
Flavonol metabolism	53
Flavone metabolism	47
Phenolic acid degradation	45
Phenylpropanoid derivative metabolism	44
Lignan biosynthesis	37
Fatty acid synthesis	31
Flavanone metabolism	24
Isoflavonoid metabolism	13
Glycerophospholipid metabolism	13
Flavan-3-ol degradation	10
Cell wall biosynthesis	8
Terpenoid backbone biosynthesis	8
Chalcone metabolism	6
Carotenoid metabolism	6
Curcuminoid metabolism	6
Arginine and proline metabolism	5
Ubiquinone and other terpenoid-quinone biosynthesis	5
Selenoamino acid metabolism	5
Stilbenoid, diarylheptanoid and gingerol biosynthesis	5
Polyketide biosynthesis	5
Steroid metabolism	4
Amino acid biosynthesis	4
Cinnamate biosynthesis	3
Caffeine metabolism	3
Ferrichrome biosynthesis	3
Coumarin metabolism	3

Supplementary Table 1. Top-30 most representative subsystems of AGREDA in comparison with AGORA. The annotation of reactions into subsystems was carried out with the Model SEED and literature. ***N*** represents the number of reactions present in AGREDA and not in AGORA annotated for a particular subsystem.

Output metabolites		AGORA		AGREDA	
		+	-	+	-
Untargeted approach	+	62	233	105	190
	-	42	119	92	69

Supplementary Table 2. Comparison of the predictions made by AGORA and AGREDA with untargeted metabolomic data for 19 metabolites captured by both approaches.

Output metabolites		AGORA		AGREDA	
		+	-	+	-
Untargeted approach	+	0	1646	1358	288
	-	0	418	382	36

Supplementary Table 3. Comparison of the predictions made by AGORA and AGREDA with untargeted metabolomic data for 86 metabolites only captured by AGREDA.

Output metabolites		AGREDA mixed-bag		AGREDA compartment	
		+	-	+	-
Untargeted approach	+	105	190	97	198
	-	92	69	77	84

Supplementary Table 4. Comparison of the predictions made by AGREDA using a mixed-bag and compartmentalized approach with untargeted metabolomic data for 19 metabolites captured by both AGORA and AGREDA.

Output metabolites		AGREDA mixed-bag		AGREDA compartment	
		+	-	+	-
Untargeted approach	+	1358	288	1347	299
	-	382	36	381	37

Supplementary Table 5. Comparison of the predictions made by AGREDA using a mixed-bag and compartmentalized approach with untargeted metabolomic data for 86 metabolites only captured by AGREDA.

Primer	Sequence
Forward	5'-TCGT CGGC AGCG TCAG ATGT GTAT AAGA GACA GCCT ACGG GNGG CWGCA-G3'
Reverse	5'-GTCT CGTG GGCT CGGA GATG TGTA TAAG AGAC AGGA CTAC HVGG GTAT CTAA TCC3'

Supplementary Table 6. Sequences of the primers employed in the 16S rRNA sequencing analysis.

Supplementary Note 1

Supplementary Figure 6 shows a toy example consisting of two species, i.e. Species 1 (beige) and Species 2 (blue). Below, this toy example is employed to illustrate the pipeline followed for the creation of AGREDA. For illustration, the reconstruction process has been divided in three steps.

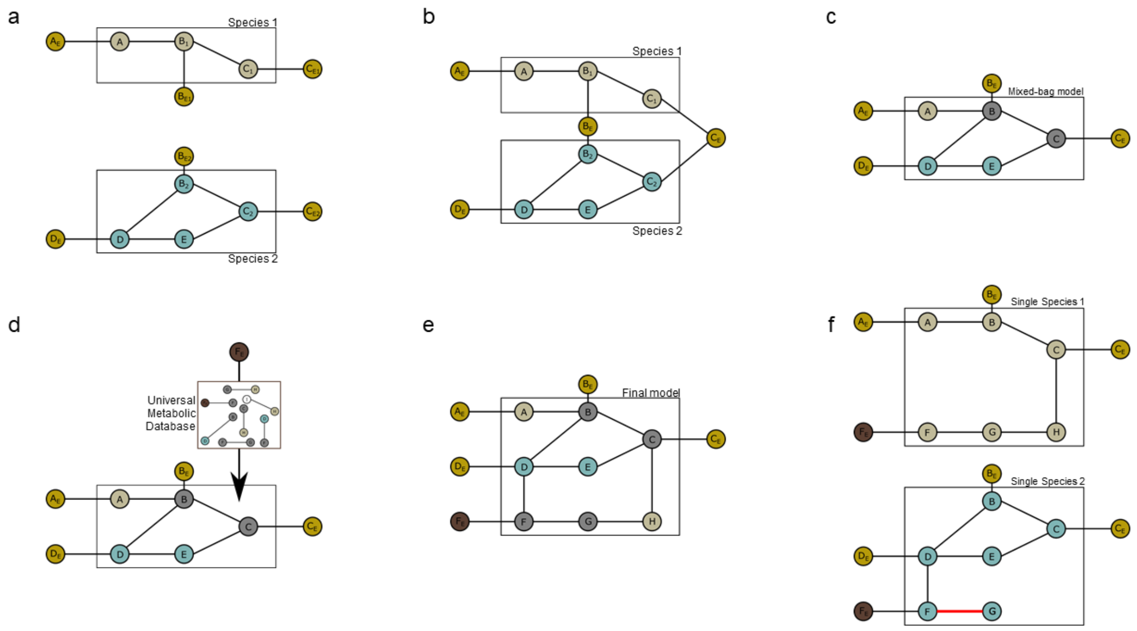

Supplementary Figure 6. Toy example for the description of the pipeline for the creation of AGREDA. (a) Toy metabolic networks of two bacterial species. **(b)** Species level metabolic model of the gut microbiota where interactions are modelled. **(c)** Resulting mixed-bag network model of the gut microbiota. **(d)** Integration of dietary compound (F_E) with the mixed-bag network model via universal metabolic databases. **(e)** Final mixed-bag metabolic model including the key diet-derived metabolites. **(f)** Single Species analysis of the final model. Beige: Species 1; Blue: Species 2; Grey: Species 1 | Species 2; Green: extracellular metabolites; Brown: key diet-derived metabolite; White: no annotation; Red: blocked reaction.

STEP 1: Modeling the microbial interactions

First, the microbial interactions must be considered and correctly modelled. This task is performed via the extracellular metabolites they share. For illustration, Supplementary Figure 6a shows that metabolites A_E and D_E are part of the metabolic networks of Species 1 and Species 2, respectively, while B_E and C_E are shared by both species. In consequence, both metabolic networks are linked through metabolites B_E and C_E, resulting in the models represented in Supplementary Figure 6b. This step was done with 818 metabolic models available in AGORA.

STEP 2: Creation of the mixed-bag network model

Next, the boundaries between the species are deleted in order to obtain a mixed-bag metabolic network model. This step consists in only keeping one per duplicated reaction, while maintaining the taxonomic assignation of the species in charge of performing the given metabolic transformation. This second step was applied to the resulting model obtained from AGORA in STEP 1.

To illustrate this step, the transformation B→C can be carried out both by Species 1 and Species 2 (Supplementary Figure 6b). In order to convert this model into the corresponding mixed-bag network model, the aforementioned duplication is removed, yielding the model shown in Supplementary Figure 6c. Notice that metabolites B and C are not beige or blue anymore, because the B→C transformation can be performed by both Species 1 and Species 2. This information is stored in the Taxonomy-Reaction (TR) rules in the same way as it is typically done with Gene-Protein-Reaction (GPR) rules in genome-scale metabolic networks. Particularly, for this reaction we have:

Species 1 | Species 2

where | represents the OR logical operator.

STEP 3: Inclusion of key metabolites and gap filling

In Step 3, dietary compounds from i-Diet which were not included in AGORA are integrated with the mixed-bag network model created in the previous step by means of the so-called universal metabolic database and gap filling techniques, obtaining the final AGREDA reconstruction.

To illustrate this step, Metabolite F_E (Supplementary Figure 6d) is a dietary compound which had not been included in the original metabolic networks shown in Supplementary Figure 6a. The gap-filling techniques described in the main manuscript are used to select a set of reactions from the universal metabolic database which link F_E to the mixed-bag model in Supplementary Figure 6c. With the aforementioned integration of dietary compounds in the mixed-bag network model, the final metabolic model of the gut microbiota is shown in Supplementary Figure 6e.

STEP 4: Single Species Analysis

This last step comprehends the assessment of blocked reactions when single-species models are extracted from the mixed-bag model in Supplementary Figure 6e.

In Supplementary Figure 6f, the single species models of Species 1 and Species 2 have been extracted from the general mixed-bag model. Although Single Species 1 presents no issues, metabolite G appears to be a dead-end metabolite in Single Species 2, in other words, F→G is a blocked reaction. In order to overcome this issue, the Single Species Analysis strategy presented in the main manuscript (Methods Section) is followed and a transport reaction for G is introduced in the final model if the suggested requirements are met.

Summary of gap filling process

Supplementary Figure 7 illustrates the gap filling process in more detail. The example metabolic pathway shown in Supplementary Figure 7a presents a gap between metabolites A and B. We aim at filling it via FastcoreWeighted, following the strategy presented in the main paper. To do so, the reactions in the universal metabolic database (Supplementary Figure 7b) shall be employed. Note that the reactions included in the universal metabolic database consist in metabolic transformations gathered from extensively used metabolic databases and bibliographic resources, which are classified in four different groups:

1. **Group 1**: Reactions assigned to AGORA species (in gray in Supplementary Figure 7b).
2. **Group 2**: Reactions not assigned to AGORA species but with functional annotation (EC numbers) (in blue in Supplementary Figure 7b).
3. **Group 3**: Reactions with neither taxonomic nor functional annotation to species in AGORA (in green in Supplementary Figure 7b).
4. **Group 4**: Reactions manually assigned to plant metabolism (in red in Supplementary Figure 7b).

The annotation of reactions to different groups is extensively detailed in the Methods section of the main text. We used available bioinformatics genome annotation tools, metabolic databases and literature knowledge.

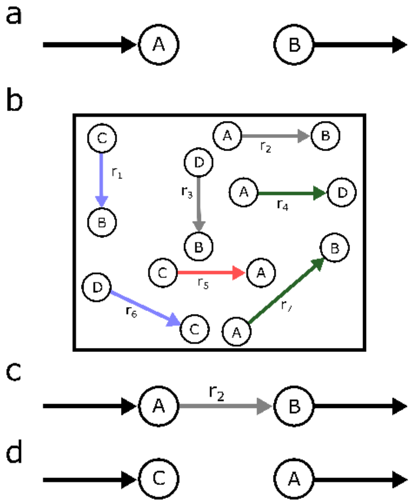

Supplementary Figure 7. Toy example of the gap filling strategy. (a) Example metabolic pathway with a gap between metabolites A and B. **(b)** Universal metabolic database. **(c)** Resulting gap-filled metabolic pathways for the example in (a). **(d)** Example metabolic pathway with a gap which cannot be filled with the reactions from the universal metabolic database in (b). Black: Core reactions; Grey: Reactions assigned to AGORA species; Blue: Reactions not assigned to AGORA species but with functional annotation; Green: Reactions with neither taxonomic nor functional annotation to species in AGORA; Red: Reactions manually assigned to plant metabolism.

A different weight is assigned to the reactions from each group in order to prioritize the inclusion of those reactions with higher confidence score. For instance, the evidence for reactions in Group 1 is higher than for the rest, since they have been assigned to AGORA species. Therefore, weights of 0.1, 50, 100 and 1000 are set to the different groups, respectively. Note that those reactions with a lower weight have a higher chance to be included in the reconstruction.

In addition, the reactions in Supplementary Figure 7a are classified as core reactions and will be forced to be part of the final model. For illustration, Supplementary Figure 7 shows that both r_2 and r_7 fit properly in the gap we aim at filling but r_2 will be selected as a solution (Supplementary Figure 7c) since it has a lower weight (higher evidence). Finally, note that if there is no candidate reaction that fits in the gap, we conclude that the given metabolite cannot be linked to the network as for the example in Supplementary Figure 7d.

References

1. Magnúsdóttir S, Heinken A, Kutt L, et al. Generation of genome-scale metabolic reconstructions for 773 members of the human gut microbiota. *Nat Biotechnol*. 2017;35(1):81-89. doi:10.1038/nbt.3703
