# Supplementary material for: An extended reconstruction of human gut microbiota metabolism of dietary compounds
Source: Nat Commun. 2021 Aug 5;12:4728. doi: 10.1038/s41467-021-25056-x (PMC8342455; doi:10.1038/s41467-021-25056-x)
Supplement: Supplementary file 3 — Description of Additional Supplementary Files [file 41467_2021_25056_MOESM3_ESM.pdf]

Description of Additional Supplementary Files

File Name: Supplementary Data 1
Description: ZIP file compressing the AGREDA mixed-bag model in MATLAB format (.MAT file).

File Name: Supplementary Data 2
Description: Excel file that includes full details about metabolites and reactions in AGREDA.

File Name: Supplementary Data 3
Description: Excel file that includes the input data used in the prediction of output microbial metabolites derived from the lentils fermentation and posterior metabolomic analyses.
