# Supplementary material for: An extended reconstruction of human gut microbiota metabolism of dietary compounds
Source: Nat Commun. 2021 Aug 5;12:4728. doi: 10.1038/s41467-021-25056-x (PMC8342455; doi:10.1038/s41467-021-25056-x)
Supplement: Supplementary file 7 — Reporting Summary [file 41467_2021_25056_MOESM7_ESM.pdf]

nature research

Corresponding author(s): M. P. Francino, I. Apaolaza, J. A. Rufián-Henares, F. J. Planes

Last updated by author(s): 12/07/2021



# Reporting Summary

Nature Research wishes to improve the reproducibility of the work that we publish. This form provides structure for consistency and transparency in reporting. For further information on Nature Research policies, see our Editorial Policies and the Editorial Policy Checklist.

## Statistics

For all statistical analyses, confirm that the following items are present in the figure legend, table legend, main text, or Methods section.

| n/a | Confirmed | |
|---|---|---|
| ☐ | ☒ | The exact sample size (*n*) for each experimental group/condition, given as a discrete number and unit of measurement |
| ☐ | ☒ | A statement on whether measurements were taken from distinct samples or whether the same sample was measured repeatedly |
| ☐ | ☒ | The statistical test(s) used AND whether they are one- or two-sided<br>*Only common tests should be described solely by name; describe more complex techniques in the Methods section.* |
| ☒ | ☐ | A description of all covariates tested |
| ☐ | ☒ | A description of any assumptions or corrections, such as tests of normality and adjustment for multiple comparisons |
| ☐ | ☒ | A full description of the statistical parameters including central tendency (e.g. means) or other basic estimates (e.g. regression coefficient) AND variation (e.g. standard deviation) or associated estimates of uncertainty (e.g. confidence intervals) |
| ☐ | ☒ | For null hypothesis testing, the test statistic (e.g. *F*, *t*, *r*) with confidence intervals, effect sizes, degrees of freedom and *P* value noted<br>*Give P values as exact values whenever suitable.* |
| ☒ | ☐ | For Bayesian analysis, information on the choice of priors and Markov chain Monte Carlo settings |
| ☒ | ☐ | For hierarchical and complex designs, identification of the appropriate level for tests and full reporting of outcomes |
| ☒ | ☐ | Estimates of effect sizes (e.g. Cohen's *d*, Pearson's *r*), indicating how they were calculated |

*Our web collection on statistics for biologists contains articles on many of the points above.*

## Software and code

Policy information about availability of computer code

| | |
|---|---|
| Data collection | No software was used to collect the data. |
| Data analysis | Matlab release R2018a, R v3.6.2, IBM Cplex v12.8, The COBRA Toolbox v3.0.0, RDKit v2020.09.1, myRAST v36, DADA2 v1.14.1, MegaBlast v2.9.0, MassLynx v4.1. |

For manuscripts utilizing custom algorithms or software that are central to the research but not yet described in published literature, software must be made available to editors and reviewers. We strongly encourage code deposition in a community repository (e.g. GitHub). See the Nature Research guidelines for submitting code & software for further information.

## Data

Policy information about availability of data

All manuscripts must include a data availability statement. This statement should provide the following information, where applicable:

- Accession codes, unique identifiers, or web links for publicly available datasets
- A list of figures that have associated raw data
- A description of any restrictions on data availability

The authors confirm that the data supporting the findings of this study are available within the article and its supplementary material. In addition the following databases have been employed throughout this study: Virtual Metabolic Human (https://www.vmh.life/), The Model SEED (https://modelseed.org/), Kyoto Encyclopedia of Genes and Genomes (https://www.genome.jp/kegg/), GenBank (https://www.ncbi.nlm.nih.gov/genbank/), Ensembl (https://www.ensembl.org/index.html), MetaCyc (https://metacyc.org/), Brenda (https://www.brenda-enzymes.org/), UniprotKB (https://www.uniprot.org/), PubChem (https://pubchem.ncbi.nlm.nih.gov/), Human Metabolome DataBase (https://hmdb.ca/), RetroRules (https://retrorules.org/), i-Diet (http://www.i-diet.es/), Phenol-Explorer (http://phenol-explorer.eu/).

# Field-specific reporting

Please select the one below that is the best fit for your research. If you are not sure, read the appropriate sections before making your selection.

- [x] Life sciences
- [ ] Behavioural & social sciences
- [ ] Ecological, evolutionary & environmental sciences

For a reference copy of the document with all sections, see nature.com/documents/nr-reporting-summary-flat.pdf

# Life sciences study design

All studies must disclose on these points even when the disclosure is negative.

| | |
|---|---|
| Sample size | We defined a limited sample size (3 children for 4 clinical conditions) in order to carry out a proof-of-concept of the predictive power of our novel gut microbiota metabolic network. A large-scale validation will be conducted in a follow-up study in the framework of STANCE4HEALTH H2020 European Project, where sample size has been exhaustively defined. |
| Data exclusions | 16S rRNA gene sequencing data belong to a broader study (within the Stance4Health project framework, EBI accession code: PRJEB40603) where the effect of using frozen vs. fresh fecal material was assessed. In order to avoid bias, we only considered frozen fecal samples, which are the commonly used in the metabolomic analyses. |
| Replication | Seven inocula were prepared with the faecal samples from lean, obese and celiac children, while six were prepared with those from children allergic to cow's milk, for a total of 27 independent fermentations. Three technical replicates were performed for targeted and untargeted metabolomic analyses. We confirm that all attempts at replication were successful. |
| Randomization | Samples were randomly assigned to different batches for 16S rRNA gene sequencing data, targeted and untargeted metabolomic analyses. |
| Blinding | The study was not a human intervention or clinical trial and, therefore, it does not require blinding for the analysis of samples. |

# Reporting for specific materials, systems and methods

We require information from authors about some types of materials, experimental systems and methods used in many studies. Here, indicate whether each material, system or method listed is relevant to your study. If you are not sure if a list item applies to your research, read the appropriate section before selecting a response.

## Materials & experimental systems

| n/a | Involved in the study | |
|---|---|---|
| x | | Antibodies |
| x | | Eukaryotic cell lines |
| x | | Palaeontology and archaeology |
| x | | Animals and other organisms |
| | x | Human research participants |
| x | | Clinical data |
| x | | Dual use research of concern |

## Methods

| n/a | Involved in the study | |
|---|---|---|
| x | | ChIP-seq |
| x | | Flow cytometry |
| x | | MRI-based neuroimaging |

## Human research participants

Policy information about studies involving human research participants

| | |
|---|---|
| Population characteristics | Age-gender: children belonging to every clinical group aged 9-11 years old. For each group 2 boys and one girl, or one boy and two girls were used.<br>Current diagnosis: lean children had a BMI >5th and <85th percentile for age, gender, and height, age, with no other diagnosed pathology in the previous 6 months. Obese children had a BMI ≥95 percentile for age, gender, and height. Celiac disease children: diagnosed celiac disease, on elimination diet (gluten free). Children allergic to cow's milk:<br>Allergy to cow's milk, on elimination diet from infancy.<br>Exclusion criteria for all the groups: GI disease (except for celiac disease children), endocrinopathies, probiotics intake last 2 weeks, antibiotics intake in the last 3 months.<br>Treatment categories: No treatment necessary to celiac disease children and those with allergy to cow's milk in the previous three months (instead elimination diet). No weight loss or other medication for obese and lean children in the previous three months. |
| Recruitment | Participants were recruited from the outpatient clinic of the Hospital Clínico (University of Granada, Spain). The survey staff described in detail to the parents of candidate participants the aim of the project and the research results that could be obtained. The parents of all the children who participated signed the relevant informed consent. |
| Ethics oversight | Informed consent was obtained from all participants in accordance with the Declaration of Helsinki. This study was approved by the Ethics Committee of the University of Granada (protocol code 1080/CEIH/2020, approved 10/06/2020). |

Note that full information on the approval of the study protocol must also be provided in the manuscript.
